# Association of Docosahexaenoic Acid and Arachidonic Acid Serum Levels With Retinopathy of Prematurity in Preterm Infants

**DOI:** 10.1001/jamanetworkopen.2021.28771

**Published:** 2021-10-14

**Authors:** Ann Hellström, Aldina Pivodic, Lotta Gränse, Pia Lundgren, Ulrika Sjöbom, Anders K. Nilsson, Helena Söderling, Anna-Lena Hård, Lois E. H. Smith, Chatarina Alice Löfqvist

**Affiliations:** 1 Department of Clinical Neuroscience, Institute of Neuroscience and Physiology, Sahlgrenska Academy, University of Gothenburg, Gothenburg, Sweden; 2 Department of Ophthalmology, Institute of Clinical Sciences Lund, Lund University and Skane University Hospital, Lund, Sweden; 3 School of Medical Sciences, Faculty of Medicine and Health, Örebro University, Örebro, Sweden; 4 Institute of Health Care Science, Sahlgrenska Academy, University of Gothenburg, Sweden; 5 Department of Ophthalmology, Boston Children's Hospital, Harvard Medical School, Boston, Massachusetts

## Abstract

**Importance:**

Supplementing preterm infants with long-chain polyunsaturated fatty acids (LC-PUFA) has been inconsistent in reducing the severity and incidence of retinopathy of prematurity (ROP). Furthermore, few studies have measured the long-term serum lipid levels after supplementation.

**Objective:**

To assess whether ROP severity is associated with serum levels of LC-PUFA, especially docosahexaenoic acid (DHA) and arachidonic acid (AA), during the first 28 postnatal days.

**Design, Setting, and Participants:**

This cohort study analyzed the Mega Donna Mega study, a randomized clinical trial that provided enteral fatty acid supplementation at 3 neonatal intensive care units in Sweden. Infants included in this cohort study were born at a gestational age of less than 28 weeks between December 20, 2016, and August 6, 2019.

**Main Outcomes and Measures:**

Severity of ROP was classified as no ROP, mild or moderate ROP (stage 1-2), or severe ROP (stage 3 and type 1). Serum phospholipid fatty acids were measured through gas chromatography–mass spectrometry. Ordinal logistic regression, with a description of unadjusted odds ratio (OR) as well as gestational age– and birth weight–adjusted ORs and 95% CIs, was used. Areas under the curve were used to calculate mean daily levels of fatty acids during postnatal days 1 to 28. Blood samples were obtained at the postnatal ages of 1, 3, 7, 14, and 28 days.

**Results:**

A total of 175 infants were included in analysis. Of these infants, 99 were boys (56.6%); the median (IQR) gestational age was 25 weeks 5 days (24 weeks 3 days to 26 weeks 6 days), and the median (IQR) birth weight was 785 (650-945) grams. A higher DHA proportion was seen in infants with no ROP compared with those with mild or moderate ROP or severe ROP (OR per 0.5–molar percentage increase, 0.49 [95% CI, 0.36-0.68]; gestational age– and birth weight–adjusted OR, 0.66 [95% CI, 0.46-0.93]). The corresponding adjusted OR for AA levels per 1–molar percentage increase was 0.83 (95% CI, 0.66-1.05). The association between DHA levels and ROP severity appeared only in infants with sufficient AA levels, suggesting that a mean daily minimum level of 7.8 to 8.3 molar percentage of AA was necessary for a detectable association between DHA level and less severe ROP.

**Conclusions and Relevance:**

This cohort study found that higher mean daily serum levels of DHA during the first 28 postnatal days were associated with less severe ROP even after adjustment for known risk factors, but only in infants with sufficiently high AA levels. Further studies are needed to identify LC-PUFA supplementation strategies that may prevent ROP and other morbidities.

## Key Points

**Question:**

Are circulatory levels and interaction of arachidonic acid (AA) and docosahexaenoic acid (DHA) associated with retinopathy of prematurity (ROP)?

**Findings:**

In this cohort study of 175 infants born at a gestational age of less than 28 weeks, higher DHA levels in the first month of life (28 postnatal days) were associated with less severe ROP but only in the presence of sufficiently high AA levels.

**Meaning:**

In this study, higher DHA levels were associated with less severe ROP in infants with sufficiently high AA levels; further studies that identify strategies for supplementation that may prevent ROP and other morbidities are warranted.

## Teaser

This cohort study evaluates the association of long-chain polyunsaturated fatty acid serum levels with the development of retinopathy of prematurity in extremely preterm infants.

## Introduction

Retinopathy of prematurity (ROP) is a neurovascular disease caused by incomplete retinal vascularization in preterm infants, with subsequent postnatal failure to complete normal vascularization. Worldwide, ROP is a major cause of childhood blindness. Given the increased rates of premature births and survival at low gestational ages with extremely immature retinal vascularization, the number of infants who are at risk for sight-threatening ROP has also increased, especially in middle-income countries.^1,2^

Extremely preterm birth cuts off the supply of the ω-6 long-chain polyunsaturated fatty acid (LC-PUFA) arachidonic acid (AA; 20:4 ω-6) and the ω-3 docosahexaenoic acid (DHA; 22:6 ω-3) from the mother. These fatty acids are structural and functional cell membrane components that are involved in energy metabolism, growth, immune defense, inflammation, vascularization, and vascular tone. They are specifically important to the eye, vascular endothelium, and brain.^3^ Experimental studies on oxygen-induced retinopathy that used a rodent ROP model reported the protective benefits of DHA-derived oxidized metabolites for sprouting angiogenesis and astrocyte survival.^4,5^ With the current neonatal care, extremely preterm infants accumulate substantial deficits in AA and DHA, which could be factors in neonatal morbidities, including ROP.^3,6,7,8^

The requirement for AA supplementation in preterm infants remains unclear. Moreover, the European Food Safety Authority recommends supplementation with DHA.^9^ Clinical trials of ω-3 LC-PUFA supplementation with fish oil that contained lipid solutions for parenteral use or with enteral DHA to prevent ROP found inconsistent results.^10,11^ One study reported an association between low serum levels of AA and ROP.^12^ Accordingly, in the multicenter Mega Donna Mega study, infants who received both AA and DHA by enteral administration from birth up to a term-equivalent age had less than half the frequency of severe ROP than infants who did not receive supplementation.^13^

We hypothesized that higher mean daily DHA and AA levels were associated with less severe ROP. Specifically, in this cohort study, we aimed to assess whether ROP severity is associated with serum levels of LC-PUFA, especially DHA and AA and their interaction, during the first 28 postnatal days.

## Methods

This cohort study was a substudy of the Mega Donna Mega study, an open-label, randomized clinical trial that was conducted at 3 neonatal intensive care units in Sweden (Queen Silvia's University Hospital, Gothenburg; Skåne University Hospital, Lund; and the Karolinska University Hospital, Stockholm). The Mega Donna Mega study was approved by the Regional Ethical Board at the University of Gothenburg, Sweden and adhered to the tenets of the Declaration of Helsinki.^14^ Written informed consent was obtained from the parents or guardians of all infants who were included in the study. Ethical approval and parent or guardian consent for the Mega Donna Mega study apply to this cohort study. We followed the Strengthening the Reporting of Observational Studies in Epidemiology (STROBE) reporting guideline.

The Mega Donna Mega study compared the outcomes of enteral supplementation with the nutritional oil Formulaid (Martek Biosciences Corporation), which provided 100 mg/kg/d of AA and 50 mg/kg/d of DHA (2:1 AA to DHA intervention group), vs no supplementation (control group) from within 3 days after birth to a postmenstrual age (PMA) of 40 weeks for severe ROP and other morbidities. Details regarding the study group, design, methods, and primary and secondary results of the Mega Donna Mega study were described by Hellström et al.^13^

### Study Participants and Procedure

**Figure 1.**
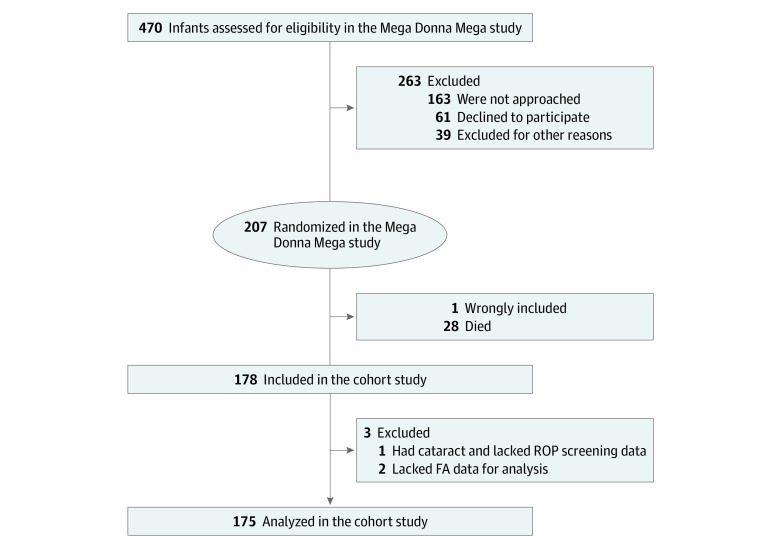
Flowchart Diagram of the Included Infants FA indicates fatty acid; ROP, retinopathy of prematurity.

A total of 190 families of 207 infants agreed to participate in the Mega Donna Mega study after providing informed consent, and these infants were randomized (Figure 1). For this cohort study, we included infants who were born at a gestational age of less than 28 weeks from December 20, 2016, to August 6, 2019. Infants with major malformations were excluded.

The nutritional strategy used in the Mega Donna Mega study has been described previously.^15^ Briefly, all infants received parenteral and enteral nutrition, following the clinical routine. Parenteral nutrition was initiated as soon as possible after birth. The parenteral lipid (ClinOleic; Baxter Healthcare) dosing strategy delivered a dose of 2 to 3 g/kg body weight at 24-hour intervals. Enteral nutrition was composed of maternal or donor breast milk with individualized fortification. Minimal enteral feeding was started within 3 hours of birth and administered at 2- to 3-hour intervals (1-2 mL/meal), with a gradual increase in volume. In the intervention group, Formulaid (0.39 mL/kg/d) was administered through enteral route from within 72 hours after birth to a PMA of 40 weeks. The dosing regimen has been described previously.^13^ The supplemented dose corresponded to the estimated fetal accretion.^13,15,16^ Infants in the control group did not receive supplementation.

### Eye Examinations and Morbidities

Screening for ROP started at a postnatal age (PNA) of 5 to 6 weeks but not before a PMA of 31 weeks. Retinal examinations through dilated pupils were performed biweekly to twice a week, depending on ROP severity. The disease was categorized according to the international classification.^17^ Regarding treatment, the Early Treatment for Retinopathy of Prematurity Cooperative Group's recommendations were followed.^18^ The outcome variable was ROP severity, which was categorized as no ROP, mild or moderate ROP (stages 1 and 2), and severe ROP (stage 3 and type 1, which indicated the requirement for ROP treatment). The screening ophthalmologists (A.H. and L.G. were 2 among other screening ophthalmologists) were blinded to the group allocation.

Prospective collection of diagnoses included bronchopulmonary dysplasia, necrotizing enterocolitis, patent ductus arteriosus, and sepsis. Bronchopulmonary dysplasia was defined as moderate-to-severe lung disease that required oxygen supplementation at a PMA of 36 weeks.^19^ Necrotizing enterocolitis was diagnosed through clinical signs and radiologic findings (Bell stages 2 and 3).^20^ Patent ductus arteriosus was recorded when the infant had clinical symptoms that required either pharmacological or surgical treatment,^21^ whereas sepsis was recorded if the infant had clinical symptoms, a C-reactive protein level higher than 20 mg/L, or an interleukin-6 level higher than 1000 ng/L along with (confirmed) or without (suspected) a positive blood culture result.^22^

### Blood Sampling and Laboratory Analyses

Whole-blood samples (0.6 mL) were collected on postnatal days 1, 3, 7, 14, and 28. Molar percentages of serum phospholipid fatty acids were identified through gas chromatography–mass spectrometry, as previously described.^13^ Data analysis was performed using MassHunter Workstation Quantitative Analysis software, version 10.0 (Agilent Technologies). Thirty-one fatty acids were quantified in all samples. The peak area of each fatty acid was normalized to that of the internal standard (19:0 methyl ester) and compared with separate linear calibration curves. Fatty acids are presented as the molar percentages of the total fatty acids analyzed. We analyzed the following LC-PUFA from the ω-6 and ω-3 series: AA (20:4 ω-6), eicosadienoic acid (20:2 ω-6), dihomo-γ-linoleic acid (20:3 ω-6), adrenic acid (22:4 ω-6), docosapentaenoic acid (22:5 ω-6), DHA (22:6 ω-3), eicosatrienoic acid (20:3 ω-3), eicosatetraenoic acid (20:4 ω-3), eicosapentaenoic acid (20:5 ω-3), and docosapentaenoic acid (22:5 ω-3).

### Statistical Analysis

The statistical methods and procedures of this cohort study were prespecified in an analysis plan. Inclusion criteria were available completed ROP screening and blood sampling results; hence, there were no missing data. The Mega Donna Mega study was powered to detect a 50% reduction of severe ROP in the intervention group vs control group. We estimated that the available sample size would suffice for the analysis of the association between the continuous LC-PUFA levels and ROP severity.

Continuous variables are presented as the mean (SD) or mean (95% CI), as appropriate, for normally distributed variables; the median (IQR) is presented for not normally distributed variables; and categorical variables are presented as number (%). The Mantel-Haenszel χ^2^ trend test was used to assess the association of dichotomous variables, whereas Jonckheere-Terpstra test was used to assess the association of continuous variables with ROP severity.

Long-chain PUFA levels were analyzed for the first month of life (PNA of 1-28 days). Longitudinal LC-PUFA levels were expressed using the individual mean area under the curve to achieve independent observations for the analysis and to calculate the mean daily molar percentages. Proportional odds ordinal logistic regression was the preferred preplanned method that allowed the assessment of more than 2 categories of ROP severity.^23^ The assumption of proportional odds was fulfilled. Therefore, binary logistic regression did not need to be continued. Given the fulfillment of proportional odds, this method estimated 1 parameter for the studied explanatory variable and c-1 intercepts, where c was the number of categories in the outcome variable. Unadjusted analyses as well as gestational age–adjusted and birth weight–adjusted analyses were performed and described using respective odds ratios (OR and adjusted OR) and 95% CIs. The estimated probability was calculated as 1/(1 + exp(−LC)), where LC was the linear combination of the respective intercept (α_i_) for mild or moderate ROP and severe ROP (with no ROP being the reference) and the obtained estimate (β_i_) multiplied by the variable value (X): LC = α_i_ + X × β_i_. Subsequently, we investigated the interaction, including an interaction term between the dichotomized mean daily AA level and continuous mean daily DHA level in the models. The outcome from these analyses was graphically presented as the continuous OR for a 0.5–molar percentage increase in DHA levels on ROP severity for levels that were lower or higher than specific AA cutoff values.

The primary analyses of the associations of the mean daily AA and DHA levels with ROP severity were planned to be confirmed by *P* < .025 in the adjusted analyses. The sensitivity analysis was performed by bootstrapping 1000 randomly selected studies (with replacement) based on the Swedish population gestational age distribution in the study by Holmström et al,^1^ describing pooled OR with 95% CI. Analyses of other LC-PUFAs were exploratory and interpreted after adjusting the transferred significance mass from the primary analyses (0.025), applying Bonferroni-Holm stepdown procedure. Given the small sample size, the interaction terms were interpreted at a *P* < .10. These analyses were considered exploratory and hypothesis-generating, predominantly interpreting the observed estimates, and were not adjusted for multiplicity.

All tests were 2-sided. For the exploratory analyses, *P* < .05 was considered statistically significant. All statistical analyses were performed using SPSS 27 for Microsoft Windows (IBM) and SAS, version 9.4 (SAS Institute Inc).

## Results

Among the 207 infants who were randomized in the Mega Donna Mega study, 178 (86.0%) survived and completed ROP screening. Those excluded from the study were 28 infants (13.5%) who did not survive the study period, and 1 infant with severe malformations who was incorrectly enrolled in the study. In addition, 1 infant with Lowe syndrome and unknown retinal status owing to congenital cataracts and 2 infants with missing blood samples for fatty acid analyses were excluded. A total of 175 infants were included in the data set (Figure 1, Table 1). Of these infants, 76 were girls (43.4%) and 99 were boys (56.6%); the median (IQR) gestational age was 25 weeks 5 days (24 weeks 3 days to 26 weeks 6 days), and the median (IQR) birth weight was 785 (650-945) grams.

**Table 1.** Clinical Characteristics of Included Infants

Characteristic	Total	ROP^**a**^			*P* value^**b**^
		No	Mild or moderate	Severe	
No. of infants (%)	175 (100)	71 (40.6)	54 (30.9)	50 (28.6)	NA
Gestational age, wk + d, median (IQR)	25 + 5 (24 + 3 to 26 + 6)	26 + 5 (26 + 1 to 27 + 3)	25 + 2 (24 + 0 to 26 + 0)	24 + 3 (24 + 0 to 25 + 4)	<.001
Median birth weight (IQR), g	785 (650 to 945)	895 (770 to 1017)	740 (600 to 925)	673 (600 to 790)	<.001
Birth weight, SDS^b^	−0.49 (−1.22 to 0.02)	−0.81 (−1.77 to −0.12)	−0.27 (−0.87 to 0.16)	−0.45 (−1.22 to 0.12)	.06
Birth weight, small for gestational age (≤2 SDS), No. (%)^b^	24 (13.7)	12 (16.9)	5 (9.3)	7 (14.0)	.57
Female sex, No. (%)	76 (43.4)	30 (42.3)	29 (53.7)	17 (34.0)	.47
Male sex, No. (%)	99 (56.6)	41 (57.7)	25 (46.3)	33 (66.0)	
BPD, No. (%)	95 (54.3)	33 (46.5)	29 (53.7)	33 (66.0)	.04
NEC, No. (%)	14 (8.0)	3 (4.2)	3 (5.6)	8 (16.0)	.02
PDA, No. (%)	93 (53.1)	20 (28.2)	36 (66.7)	37 (74.0)	<.001
Sepsis, No. (%)	85 (48.6)	30 (42.3)	25 (46.3)	30 (60.0)	.06

Abbreviations: BPD, bronchopulmonary dysplasia; NA, not applicable; NEC, necrotizing enterocolitis; PDA, patent ductus arteriosus; ROP, retinopathy of prematurity; SDS, standard deviation score.

^a^ For between-group comparisons, the Mantel-Haenszel χ^2^ test and the Jonckheere-Terpstra test were used for ordered categorical and continuous variables, respectively.

^b^ The applied level of significance was *P* < .05.

Among the participants, 71 (40.6%) had no ROP, 54 (30.9%) developed mild or moderate ROP (stage 1 to 2) at a mean (95% CI) PNA of 9.5 (8.9-10.1) weeks, and 50 (28.6%) had severe ROP (stage 3 or type 1) at a mean (95% CI) PNA of 12.4 (11.6-13.3) weeks. A total of 35 infants (20.0%) were treated for sight-threatening ROP at a mean (95% CI) PNA of 12.3 (11.4-13.2) weeks. Table 1 summarizes the clinical characteristics of the infants overall and according to the ROP severity.

### DHA and AA Serum Levels and ROP Severity

The primary hypothesis was that lower mean daily DHA and AA levels during the first 28 postnatal days were associated with severe ROP (ie, higher stages). The hypothesis was confirmed for DHA level (OR per 0.5–molar percentage increase, 0.49 [95% CI, 0.36-0.68]; gestational age– and birth weight–adjusted OR, 0.66 [95% CI, 0.46-0.93]), but not for AA level (adjusted OR per 1–molar percentage increase, 0.83; 95% CI, 0.66-1.05) (eTable 1 in the Supplement). The adjusted results for DHA and AA were confirmed in the sensitivity analysis using bootstrapping, applying gestational age distribution according to the Swedish population (pooled adjusted OR per 0.5–molar percentage increase, 0.64 [95% CI, 0.43-0.95]; pooled adjusted OR per 1–molar percentage increase, 0.82 [95% CI, 0.64-1.05]).

**Figure 2.**
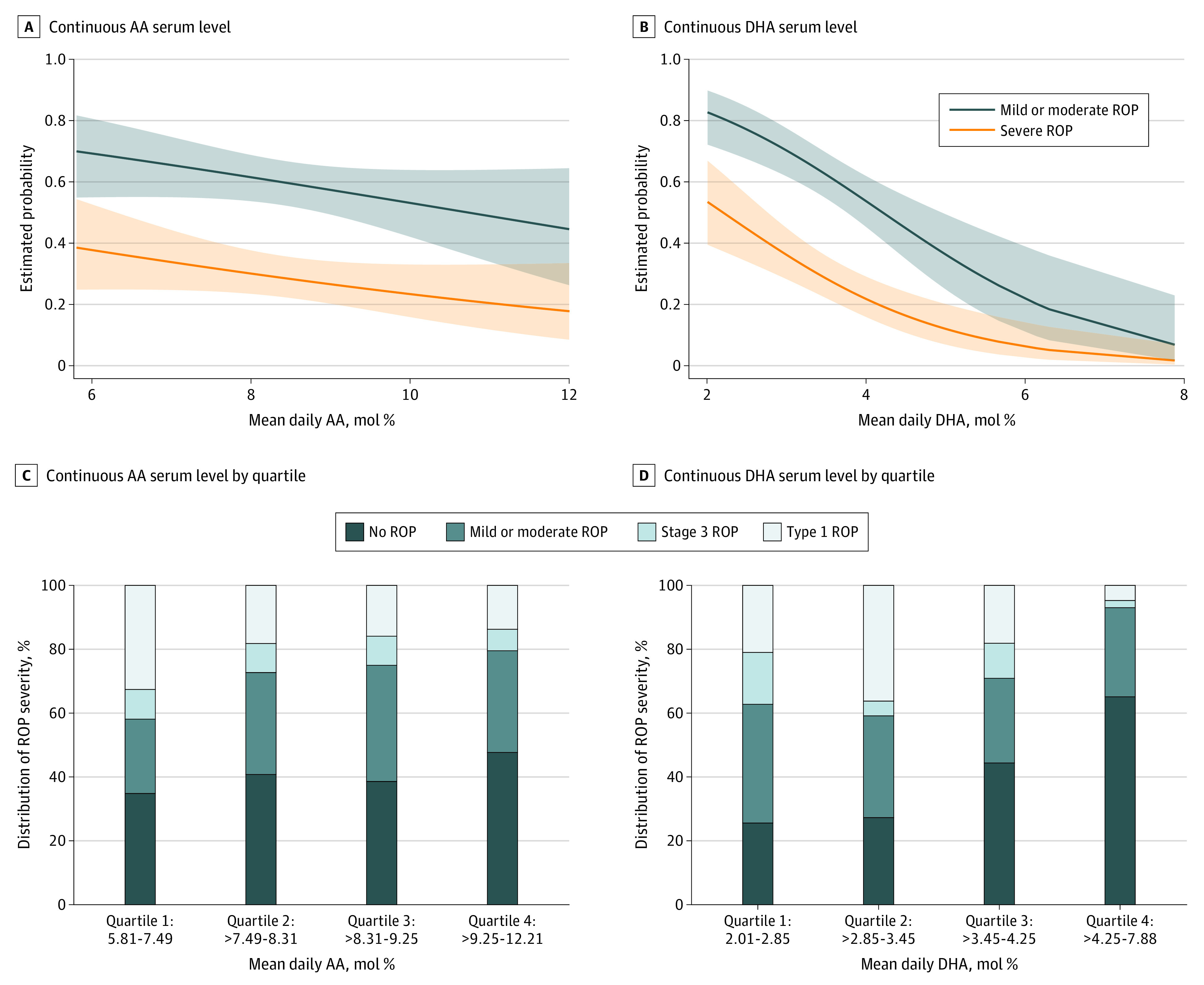
Probability Plots and Percentage Distribution of Retinopathy of Prematurity (ROP) Severity for Continuous Arachidonic Acid (AA) and Docosahexaenoic Acid (DHA) Serum Levels Mild or moderate ROP includes stages 1 and 2 (nontreated). The shaded areas in panels A and B indicate the 95% CIs.

Figure 2 A and B shows the probability plots of ROP severity for continuous AA and DHA serum levels, respectively, and Figure 2 C and D shows the percentage distribution of ROP severity for continuous AA and DHA serum levels categorized into quartiles, respectively. In these figures, the associations between higher levels of fatty acids (DHA in particular) and less severe ROP were visible.

**Figure 3.**
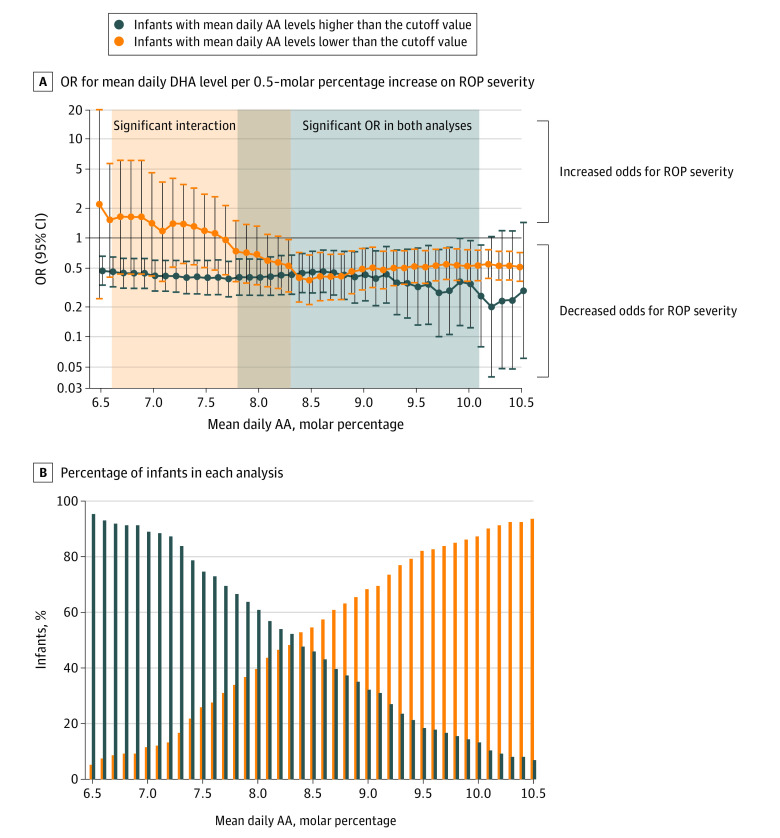
Odds Ratio (OR) for Retinopathy of Prematurity (ROP) Severity According to Arachidonic Acid (AA) Dichotomized at Different Cutoff Values and Interaction With Continuous Docosahexaenoic Acid (DHA) A, The ORs represent the mean daily DHA per 0.5–molar percentage increase on ROP severity for infants with lower values than a certain AA cutoff and for infants with higher values than a certain AA cutoff. Each AA cutoff value corresponds with 2 obtained ORs: lower (orange curve) and higher (blue curve) than the cutoff value. An OR of 0.4 indicates a 60% decrease in the odds for ROP progression between severe ROP and mild or moderate ROP and between mild or moderate ROP and no ROP. The orange field indicates a significant association (*P* < .10) between the results for the orange and blue curves. The gray field indicates the suggested minimum AA level at which the DHA level would be significantly associated with ROP severity. The blue field indicates where both the orange and blue curves show a significant association between higher DHA levels and less severe ROP. B, The percentage of infants in each analysis are presented in the needle graph.

We performed in-depth analyses to ascertain the lowest AA cutoff value at which higher DHA levels were associated with less severe ROP. Those analyses included interactions between various AA molar percentage categories (dichotomized along the AA scale) and continuous DHA levels. Parameter estimates for these interactions were significant for AA cutoff values that were lower than 7.8 molar percentage, which was interpreted as follows: the association between DHA level and ROP severity significantly differed for infants with AA levels that were lower vs higher than a certain cutoff value (Figure 3; eTable 2 in the Supplement). In the analyses of infants with AA levels that were lower than the 7.8 molar percentage cutoff value, the OR was close to 1.00 compared with the estimated OR of less than 0.50 among infants with AA levels that were higher than the cutoff value. In addition, for AA cutoff values that were higher than or equal to 8.3 molar percentage, the association between higher DHA levels and less severe ROP was significant for subgroups with mean daily AA levels that were lower or higher than certain cutoff values. This finding suggested that the mean daily AA level of at least 7.8 to 8.3 molar percentage was required for an association between higher DHA level and less severe ROP. Analyses that adjusted for gestational age and birth weight showed similar but nonsignificant patterns with attenuated estimates and wider CIs (eTable 3 and eFigure in the Supplement).

### Other LC-PUFA Levels and ROP Severity

Other LC-PUFAs of both the ω-6 and ω-3 series with lower levels than DHA were associated with more severe ROP in unadjusted analyses after a Bonferroni-Holm adjustment for multiplicity. These LC-PUFAs included eicosadienoic acid (OR, 0.38; 95% CI, 0.25-0.59), dihomo-γ-linoleic acid (OR, 0.42; 95% CI, 0.25-0.70), eicosatrienoic acid (OR, 0.51; 95% CI, 0.38-0.70), and eicosatetraenoic acid (OR, 0.76; 95% CI, 0.65-0.89). Lower mean daily levels (PNA days 1-28) of eicosadienoic acid (OR, 0.45; 95% CI, 0.28-0.71) and eicosatrienoic acid (OR, 0.62; 95% CI, 0.45-0.86) were associated with more severe ROP, even after adjustment for gestational age and birth weight, applying Bonferroni-Holm (Table 2; eTable 1 in the Supplement).

**Table 2.** Median Daily Long-Chain Polyunsaturated Fatty Acid Levels During the First 28 Postnatal Days in Infants With No, Mild or Moderate, or Severe Retinopathy of Prematurity (ROP)

Fatty acid common name	Median area under the curve (IQR), molar percentage		
	No ROP (n = 71)	Mild or moderate ROP (n = 54)	Severe ROP (n = 50)
ω-3			
20:3 ω-3 (Eicosatrienoic acid)	0.03 (0.02-0.04)	0.02 (0.02-0.03)	0.02 (0.02-0.03)
20:4 ω-3 (Eicosatetraenoic acid)	0.04 (0.03-0.06)	0.03 (0.03-0.05)	0.03 (0.02-0.04)
20:5 ω-3 (Eicosapentaenoic acid)	0.52 (0.38-0.63)	0.42 (0.35-0.49)	0.42 (0.31-0.56)
22:5 ω-3 (Docosapentaenoic acid)	0.12 (0.10-0.16)	0.11 (0.09-0.14)	0.12 (0.10-0.13)
22:6 ω-3 (DHA)	1.93 (1.64-2.42)	1.69 (1.41-2.10)	1.54 (1.32-1.85)
ω-6			
20:2 ω-6 (Eicosadienoic acid)	0.31 (0.26-0.37)	0.28 (0.24-0.32)	0.25 (0.21-0.30)
20:3 ω-6 (Dihomo-γ-linolenic acid)	2.48 (2.07-2.86)	2.16 (1.92-2.63)	2.07 (1.73-2.43)
20:4 ω-6 (AA)	8.35 (7.55-9.55)	8.57 (7.72-9.29)	8.09 (7.34-8.97)
22:4 ω-6 (Adrenic acid)	0.08 (0.07-0.09)	0.08 (0.07-0.09)	0.08 (0.07-0.09)
22:5 ω-6 (Docosapentaenoic acid)	0.10 (0.08-0.13)	0.10 (0.08-0.12)	0.10 (0.09-0.13)

Abbreviations: AA, arachidonic acid; DHA, docosahexaenoic acid.

## Discussion

In this cohort, higher mean daily serum levels of DHA, but not significantly higher levels of AA, during the first 28 postnatal days were associated with less severe ROP. These findings suggested that a minimum mean daily AA serum level of 7.8 to 8.3 molar percentage was required to achieve the association between higher DHA level and less severe ROP. Interaction analyses of AA and DHA levels and ROP severity that adjusted for gestational age and birth weight showed similar but not significant results.

The roles of LC-PUFA in the first ROP phase remain unclear. Oxidative stress from deranged oxygenation, mechanical ventilation, parenteral nutrition, sepsis, blood transfusions, and other factors are crucially involved in ROP development.^24^ Both AA and DHA are highly unsaturated fatty acids and prone to peroxidation. However, proper membrane inclusion of these LC-PUFAs could promote membrane integrity and prevent oxidative stress damage.^3^ Greater availability of AA and DHA may increase membranes' integrity in the central nervous system and vasculature.^3^

During gestation, there is a selective transfer of AA and DHA from the mother to the fetus.^25^ From 24 weeks’ gestation, AA levels in fetal blood are approximately double those in maternal blood. The fetal DHA fractions are similar to those of the mother until approximately 30 weeks’ gestation when they sharply increase concomitantly with the brain growth surge.^26^ Although maternal DHA levels and the placental transfer to the fetus are dependent on the mother’s diet, AA levels appear to be less diet dependent.^27^ Western diets commonly result in low DHA levels in pregnant women. In the case of high maternal levels, the transplacental DHA transport is limited, resulting in bioattenuation.^28,29^ Preterm infants can also synthesize AA and DHA from their precursors, linoleic acid and α-linolenic acid, respectively.^30,31^

High levels of the ω-3 DHA and eicosapentaenoic acid can suppress AA levels. Accordingly, supplementation with ω-3 fatty acids from fish oil in preterm infants can decrease AA serum levels.^32,33^ A previous study reported an association between decreased AA serum levels during the first postnatal month and ROP.^12^ Lower or higher doses of ω-3 LC-PUFA might increase or decrease membrane AA levels.^34^

A need for PMA-dependent supplementation has been suggested given that the AA or DHA quotient decreases during gestation from a ratio of 4.9 at 24 to 27 weeks’ gestation to 2.5 at term; moreover, low AA or DHA ratios before a PMA of 28 weeks are associated with bronchopulmonary dysplasia severity.^26^ Without AA supplementation, the DINO (DHA for the Improvement of Neurodevelopmental Outcome in Preterm Infants) trial on DHA supplementation from 3 days after birth until a PMA of 36 weeks or discharge in infants with gestational age of less than 29 weeks found no association with or a possibly harmful outcome for lung development.^11^ In term infants, previous studies have reported a bell-shaped curve of red blood cell AA levels with increasing eicosapentaenoic acid or DHA^35^ and DHA^36^ levels, as well as a bell-shaped curve of intelligence quotient with worse outcome when the DHA fraction exceeded the AA fraction.^36^ Very preterm infants who received formula with 2:1 AA to DHA during their first year of life had better psychomotor development than infants who received formula with 1:1 AA to DHA.^37^

Given the importance of AA in growth and development, especially of the central nervous system, providing AA supplementation to the extremely preterm newborn is likely needed to compensate for maternal supply loss. However, it is essential to balance AA and DHA or eicosapentaenoic acid levels to favor the physiological incorporation of these fatty acids. If future studies on dosage and short- and long-term outcomes of AA and DHA supplementation agree with the results, about 50% of infants with ROP who need treatment might be avoided.^13^ Thus, many premature infants may be spared 1 or repeated laser treatment sessions with general anesthesia or intravitreal injections of anti–vascular endothelial growth factor drugs. This may substantially change their long-term health given the increasing concerns about the adverse effects on the preterm brain of general anesthesia and anti–vascular endothelial growth factor injections.^38^

### Strengths and Limitations

This study has some strengths. A strength of this study is the use of ordinal logistic regression to analyze ROP severity, which mimics the disease's pathophysiological processes.^23^ Unlike binary logistic regression, this method allowed more than 2 categories to be studied for the outcome variable, provided that the assumption of proportional odds was fulfilled. Another strength is the study's prospective design and the use of standardized protocols in screening, classification, and ROP diagnosis that reduced information bias.

This study also has several limitations. The study population was limited to infants with gestational age of less than 28 weeks, suggesting a potential selection bias because of the generalization of the results to all infants who were at risk for ROP. Although the results were confirmed in the sensitivity analysis, future external validations can fully evaluate the generalizability of the observed results. The sample size was not sufficiently large to enable adjusted exploratory interaction analyses to confirm the unadjusted results of the association of AA and DHA levels with ROP severity, although the estimates suggested similar results. In addition, the sample size was small for the context of adjusting for multiplicity issues in the interaction analysis (which generally requires large data sets), why they should be evaluated as highly exploratory and rather hypotheses generating.

## Conclusions

This cohort study found that higher serum DHA levels during the first 28 postnatal days were associated with less severe ROP even after adjustment for known risk factors, but there was no association for AA levels. The association between higher DHA level and less severe ROP appeared to emerge with sufficiently high AA levels. Therefore, further studies of nutrition strategies are needed to identify LC-PUFA associations that may prevent ROP and possibly other morbidities.

## Notes

Supplement. **eTable 1.** Unadjusted and Adjusted Ordinal Logistic Regression for ROP Severity Explained by Long-Chain Polyunsaturated Fatty Acids **eTable 2.** Unadjusted Ordinal Logistic Regression for ROP Severity Studying Interaction Between Continuous DHA and Dichotomous AA (Below and Above Different Cut-offs) **eTable 3.** Gestational Age and Birth Weight Adjusted Ordinal Logistic Regression for ROP Severity Studying Interaction Between Continuous DHA and Dichotomous AA (Below and Above Different Cut-offs) **eFigure.** Gestational Age and Birth Weight Adjusted Odds-Ratios Obtained from the Ordinal Logistic Regression for ROP Severity Studying Interaction Between Continuous DHA and Dichotomous AA (Below and Above Different Cut-offs)

## References

[1] Holmström G, Hellström A, Gränse L, . New modifications of Swedish ROP guidelines based on 10-year data from the SWEDROP register. Br J Ophthalmol. 2020;104(7):943-949. doi:10.1136/bjophthalmol-2019-314874 31676594

[2] Blencowe H, Moxon S, Gilbert C. Update on blindness due to retinopathy of prematurity globally and in India. Indian Pediatr. 2016;53(suppl 2):S89-S92. 27915313

[3] Crawford MA, Golfetto I, Ghebremeskel K, . The potential role for arachidonic and docosahexaenoic acids in protection against some central nervous system injuries in preterm infants. Lipids. 2003;38(4):303-315. doi:10.1007/s11745-003-1065-1 12848275

[4] Sapieha P, Stahl A, Chen J, . 5-lipoxygenase metabolite 4-HDHA is a mediator of the antiangiogenic effect of ω-3 polyunsaturated fatty acids. Sci Transl Med. 2011;3(69):69ra12. doi:10.1126/scitranslmed.3001571 21307302 PMC3711031

[5] Hu J, Bibli SI, Wittig J, . Soluble epoxide hydrolase promotes astrocyte survival in retinopathy of prematurity. J Clin Invest. 2019;129(12):5204-5218. doi:10.1172/JCI123835 31479425 PMC6877309

[6] Martin CR, Dasilva DA, Cluette-Brown JE, . Decreased postnatal docosahexaenoic and arachidonic acid blood levels in premature infants are associated with neonatal morbidities. J Pediatr. 2011;159(5):743-749.e1, 2. doi:10.1016/j.jpeds.2011.04.039 21658712 PMC3701520

[7] Böckmann KA, von Stumpff A, Bernhard W, . Fatty acid composition of adipose tissue at term indicates deficiency of arachidonic and docosahexaenoic acid and excessive linoleic acid supply in preterm infants. Eur J Nutr. 2021;60(2):861-872. doi:10.1007/s00394-020-02293-2 32476053 PMC7900037

[8] De Rooy L, Hamdallah H, Dyall SC. Extremely preterm infants receiving standard care receive very low levels of arachidonic and docosahexaenoic acids. Clin Nutr. 2017;36(6):1593-1600. doi:10.1016/j.clnu.2016.09.033 27756480

[9] Crawford MA, Wang Y, Forsyth S, Brenna JT. The European Food Safety Authority recommendation for polyunsaturated fatty acid composition of infant formula overrules breast milk, puts infants at risk, and should be revised. Prostaglandins Leukot Essent Fatty Acids. 2015;102-103:1-3. doi:10.1016/j.plefa.2015.07.005 26432509

[10] Bernabe-García M, Villegas-Silva R, Villavicencio-Torres A, . Enteral docosahexaenoic acid and retinopathy of prematurity: a randomized clinical trial. JPEN J Parenter Enteral Nutr. 2019;43(7):874-882. doi:10.1002/jpen.1497 30614004

[11] Collins CT, Makrides M, McPhee AJ, . Docosahexaenoic acid and bronchopulmonary dysplasia in preterm infants. N Engl J Med. 2017;376(13):1245-1255. doi:10.1056/NEJMoa1611942 28355511

[12] Löfqvist CA, Najm S, Hellgren G, . Association of retinopathy of prematurity with low levels of arachidonic acid: a secondary analysis of a randomized clinical trial. JAMA Ophthalmol. 2018;136(3):271-277. doi:10.1001/jamaophthalmol.2017.6658 29423508 PMC5885898

[13] Hellström A, Nilsson AK, Wackernagel D, . Effect of enteral lipid supplement on severe retinopathy of prematurity: a randomized clinical trial. JAMA Pediatr. 2021;175(4):359-367. doi:10.1001/jamapediatrics.2020.5653 33523106 PMC7851754

[14] World Medical Association. World Medical Association Declaration of Helsinki: ethical principles for medical research involving human subjects. JAMA. 2013;310(20):2191-2194. doi:10.1001/jama.2013.281053 24141714

[15] Lapillonne A, Groh-Wargo S, Gonzalez CH, Uauy R. Lipid needs of preterm infants: updated recommendations. J Pediatr. 2013;162(3 suppl):S37-S47. doi:10.1016/j.jpeds.2012.11.052 23445847

[16] Kuipers RS, Luxwolda MF, Offringa PJ, Boersma ER, Dijck-Brouwer DA, Muskiet FA. Fetal intrauterine whole body linoleic, arachidonic and docosahexaenoic acid contents and accretion rates. Prostaglandins Leukot Essent Fatty Acids. 2012;86(1-2):13-20. doi:10.1016/j.plefa.2011.10.012 22115845

[17] International Committee for the Classification of Retinopathy of Prematurity. The international classification of retinopathy of prematurity revisited. Arch Ophthalmol. 2005;123(7):991-999. doi:10.1001/archopht.123.7.991 16009843

[18] Early Treatment for Retinopathy of Prematurity Cooperative Group. Revised indications for the treatment of retinopathy of prematurity: results of the early treatment for retinopathy of prematurity randomized trial. Arch Ophthalmol. 2003;121(12):1684-1694. doi:10.1001/archopht.121.12.1684 14662586

[19] Haggie S, Robinson P, Selvadurai H, Fitzgerald DA. Bronchopulmonary dysplasia: a review of the pulmonary sequelae in the post-surfactant era. J Paediatr Child Health. 2020;56(5):680-689. doi:10.1111/jpc.14878 32270551

[20] Juhl SM, Hansen ML, Gormsen M, Skov T, Greisen G. Staging of necrotising enterocolitis by Bell’s criteria is supported by a statistical pattern analysis of clinical and radiological variables. Acta Paediatr. 2019;108(5):842-848. doi:10.1111/apa.14469 29926969

[21] Park J, Yoon SJ, Han J, . Patent ductus arteriosus treatment trends and associated morbidities in neonates. Sci Rep. 2021;11(1):10689. doi:10.1038/s41598-021-89868-z 34021202 PMC8139968

[22] Mirzarahimi M, Barak M, Eslami A, Enteshari-Moghaddam A. The role of interleukin-6 in the early diagnosis of sepsis in premature infants. Pediatr Rep. 2017;9(3):7305. doi:10.4081/pr.2017.7305 29081936 PMC5643948

[23] Harrell FE Jr. Regression Modeling Strategies. With Applications to Linear Models, Logistic and Ordinal Regression, and Survival Analysis. Springer International Publishing; 2015.

[24] Graziosi A, Perrotta M, Russo D, . Oxidative stress markers and the retinopathy of prematurity. J Clin Med. 2020;9(9):2711. doi:10.3390/jcm9092711 32825796 PMC7563779

[25] Haggarty P. Fatty acid supply to the human fetus. Annu Rev Nutr. 2010;30:237-255. doi:10.1146/annurev.nutr.012809.104742 20438366

[26] Bernhard W, Raith M, Koch V, . Developmental changes in polyunsaturated fetal plasma phospholipids and feto-maternal plasma phospholipid ratios and their association with bronchopulmonary dysplasia. Eur J Nutr. 2016;55(7):2265-2274. doi:10.1007/s00394-015-1036-5 26363610

[27] Makrides M, Neumann MA, Byard RW, Simmer K, Gibson RA. Fatty acid composition of brain, retina, and erythrocytes in breast- and formula-fed infants. Am J Clin Nutr. 1994;60(2):189-194. doi:10.1093/ajcn/60.2.189 7913291

[28] Kuipers RS, Luxwolda MF, Sango WS, Kwesigabo G, Dijck-Brouwer DA, Muskiet FA. Maternal DHA equilibrium during pregnancy and lactation is reached at an erythrocyte DHA content of 8 g/100 g fatty acids. J Nutr. 2011;141(3):418-427. doi:10.3945/jn.110.128488 21270355

[29] Luxwolda MF, Kuipers RS, Sango WS, Kwesigabo G, Dijck-Brouwer DA, Muskiet FA. A maternal erythrocyte DHA content of approximately 6 g% is the DHA status at which intrauterine DHA biomagnifications turns into bioattenuation and postnatal infant DHA equilibrium is reached. Eur J Nutr. 2012;51(6):665-675. doi:10.1007/s00394-011-0245-9 21952690 PMC3419349

[30] Salem N Jr, Wegher B, Mena P, Uauy R. Arachidonic and docosahexaenoic acids are biosynthesized from their 18-carbon precursors in human infants. Proc Natl Acad Sci U S A. 1996;93(1):49-54. doi:10.1073/pnas.93.1.49 8552667 PMC40176

[31] Carnielli VP, Wattimena DJ, Luijendijk IH, Boerlage A, Degenhart HJ, Sauer PJ. The very low birth weight premature infant is capable of synthesizing arachidonic and docosahexaenoic acids from linoleic and linolenic acids. Pediatr Res. 1996;40(1):169-174. doi:10.1203/00006450-199607000-00029 8798265

[32] Najm S, Löfqvist C, Hellgren G, . Effects of a lipid emulsion containing fish oil on polyunsaturated fatty acid profiles, growth and morbidities in extremely premature infants: a randomized controlled trial. Clin Nutr ESPEN. 2017;20:17-23. doi:10.1016/j.clnesp.2017.04.004 29072164 PMC5784264

[33] D’Ascenzo R, Savini S, Biagetti C, . Higher docosahexaenoic acid, lower arachidonic acid and reduced lipid tolerance with high doses of a lipid emulsion containing 15% fish oil: a randomized clinical trial. Clin Nutr. 2014;33(6):1002-1009. doi:10.1016/j.clnu.2014.01.009 24525115

[34] Horrobin DF, Jenkins K, Bennett CN, Christie WW. Eicosapentaenoic acid and arachidonic acid: collaboration and not antagonism is the key to biological understanding. Prostaglandins Leukot Essent Fatty Acids. 2002;66(1):83-90. doi:10.1054/plef.2001.0338 12051959

[35] Luxwolda MF, Kuipers RS, Smit EN, Velzing-Aarts FV, Dijck-Brouwer DA, Muskiet FA. The relation between the omega-3 index and arachidonic acid is bell shaped: synergistic at low EPA+DHA status and antagonistic at high EPA+DHA status. Prostaglandins Leukot Essent Fatty Acids. 2011;85(3-4):171-178. doi:10.1016/j.plefa.2011.05.004 21715149

[36] Colombo J, Jill Shaddy D, Kerling EH, Gustafson KM, Carlson SE. Docosahexaenoic acid (DHA) and arachidonic acid (ARA) balance in developmental outcomes. Prostaglandins Leukot Essent Fatty Acids. 2017;121:52-56. doi:10.1016/j.plefa.2017.05.005 28651697 PMC5819348

[37] Alshweki A, Muñuzuri AP, Baña AM, . Effects of different arachidonic acid supplementation on psychomotor development in very preterm infants; a randomized controlled trial. Nutr J. 2015;14:101. doi:10.1186/s12937-015-0091-3 26424477 PMC4590272

[38] Tan H, Blasco P, Lewis T, Ostmo S, Chiang MF, Campbell JP. Neurodevelopmental outcomes in preterm infants with retinopathy of prematurity. Surv Ophthalmol. 2021;66(5):877-891. doi:10.1016/j.survophthal.2021.02.012 33667496 PMC8351023

