# Supplementary material for: Association of Docosahexaenoic Acid and Arachidonic Acid Serum Levels With Retinopathy of Prematurity in Preterm Infants
Source: JAMA Netw Open. 2021 Oct 14;4(10):e2128771. doi: 10.1001/jamanetworkopen.2021.28771 (PMC8517742; doi:10.1001/jamanetworkopen.2021.28771)
Supplement: Supplement. — eTable 1. Unadjusted and Adjusted Ordinal Logistic Regression for ROP Severity Explained by Long-Chain Polyunsaturated Fatty Acids eTable 2. Unadjusted Ordinal Logistic Regression for ROP Severity Studying Interaction Between Continuous DHA and Dichotomous AA (Below and Above Different Cut-offs) eTable 3. Gestational Age and Birth Weight Adjusted Ordinal Logistic Regression for ROP Severity Studying Interaction Between Continuous DHA and Dichotomous AA (Below and Above Different Cut-offs) eFigure. Gestational Age and Birth Weight Adjusted Odds-Ratios Obtained from the Ordinal Logistic Regression for ROP Severity Studying Interaction Between Continuous DHA and Dichotomous AA (Below and Above Different Cut-offs) [file jamanetwopen-e2128771-s001.pdf]

Supplementary Online Content

Hellström A, Pivodic A, Gränse L, et al. Association of docosahexaenoic acid and arachidonic acid serum levels with retinopathy of prematurity in preterm infants. *JAMA Netw Open*. 2021;4(10):e2128771. doi:10.1001/jamanetworkopen.2021.28771

eTable 1. Unadjusted and Adjusted Ordinal Logistic Regression for ROP Severity Explained by Long-Chain Polyunsaturated Fatty Acids

eTable 2. Unadjusted Ordinal Logistic Regression for ROP Severity Studying Interaction Between Continuous DHA and Dichotomous AA (Below and Above Different Cut-offs)

eTable 3. Gestational Age and Birth Weight Adjusted Ordinal Logistic Regression for ROP Severity Studying Interaction Between Continuous DHA and Dichotomous AA (Below and Above Different Cut-offs)

eFigure. Gestational Age and Birth Weight Adjusted Odds-Ratios Obtained from the Ordinal Logistic Regression for ROP Severity Studying Interaction Between Continuous DHA and Dichotomous AA (Below and Above Different Cut-offs)

This supplementary material has been provided by the authors to give readers additional information about their work.

© 2021 Hellström A et al. *JAMA Network Open.*

eTable 1. Unadjusted and Adjusted Ordinal Logistic Regression for ROP Severity Explained by Long-Chain Polyunsaturated Fatty Acids

	Ordinal logistic regression for ROP severity	
	OR (95% CI)<br>p-value<br>p-value for proportional odds assumption	
**Variable**	**Unadjusted**	**Adjusted for GA and weight**
**Mean AUC day 1-28 20:4 ω-6 mol% (arachidonic acid)<br>(OR per 1 mol% increase)**	0.84 (0.68 - 1.04)<br>p=0.12<br>p prop. = 0.45	0.83 (0.66 - 1.05)<br>p=0.13<br>p prop. = 0.06
**Mean AUC day 1-28 22:6 ω-3 mol% (docosahexaenoic acid)<br>(OR per 0.5 mol% increase)**	0.49 (0.36 - 0.68)<br>p<0.001 (0.025 sign.)<br>p prop. = 0.67	0.66 (0.46 - 0.93)<br>p=0.02 (0.025 sign.)<br>p prop. = 0.06
**Mean AUC day 1-28 20:2 ω-6 mol% (eicosadienoic acid)<br>(OR per 0.1 mol% increase)**	0.38 (0.25 - 0.59)<br>p<0.001 (Holm sign.)<br>p prop. = 0.82	0.45 (0.28 - 0.71)<br>p<0.001 (Holm sign.)<br>p prop. = 0.05
**Mean AUC day 1-28 20:3 ω-6 mol% (dihomo y-linoleic acid)<br>(OR per 1 mol% increase)**	0.42 (0.25 - 0.70)<br>p=0.001 (Holm sign.)<br>p prop. = 0.64	0.60 (0.34 - 1.04)<br>p=0.07<br>p prop. = 0.05
**Mean AUC day 1-28 22:4 ω-6 mol% (adrenic acid)<br>(OR per 0.01 mol% increase)**	0.95 (0.80 - 1.14)<br>p=0.61<br>p prop. = 0.15	1.03 (0.85 - 1.26)<br>p=0.76<br>p prop. = 0.03
**Mean AUC day 1-28 22:5 ω-6 mol% (docosapenatenoic acid)<br>(OR per 0.01 mol% increase)**	1.04 (0.96 - 1.12)<br>p=0.34<br>p prop. = 0.10	1.09 (1.00 - 1.19)<br>p=0.05<br>p prop. = 0.03
**Mean AUC day 1-28 20:3 ω-3 mol% (eicosatrienoic acid)<br>(OR per 0.01 mol% increase)**	0.51 (0.38 - 0.70)<br>p<0.001 (Holm sign.)<br>p prop. = 0.38	0.62 (0.45 - 0.86)<br>p=0.004 (Holm sign.)<br>p prop. = 0.04
**Mean AUC day 1-28 20:4 ω-3 mol% (eicosatetraenoic acid)<br>(OR per 0.01 mol% increase)**	0.76 (0.65 - 0.89)<br>p<0.001 (Holm sign.)<br>p prop. = 0.57	0.86 (0.72 - 1.01)<br>p=0.07<br>p prop. = 0.06
**Mean AUC day 1-28 20:5 ω-3 mol% (eicosapentaenoic acid)<br>(OR per 0.1 mol% increase)**	0.86 (0.75 - 1.00)<br>p=0.05<br>p prop. = 0.14	0.94 (0.81 - 1.09)<br>p=0.43<br>p prop. = 0.02
**Mean AUC day 1-28 22:5 ω-3 mol% (docasapentaenoic acid)<br>(OR per 0.1 mol% increase)**	0.45 (0.21 - 0.94)<br>p=0.03<br>p prop. = 0.85	0.84 (0.37 - 1.90)<br>p=0.68<br>p prop. = 0.08

ROP = retinopathy of prematurity; LCPUFAs = long-chain polyunsaturated fatty acid; OR = odds ratio; CI = confidence interval; AUC = area under the curve

The confirmatory primary analyses for arachidonic acid and docosahexaenoic acid is p<0.025. Significant result was denoted by (0.025 sign.).
For all other variables Bonferroni-Holm stepdown procedure was applied on 0.025 significance level. Significant results were denoted by (Holm sign.).
The level of significance for the proportional odds assumption is p<0.05.

© 2021 Hellström A et al. *JAMA Network Open.*

eTable 2. Unadjusted Ordinal Logistic Regression for ROP Severity Studying Interaction Between Continuous DHA and Dichotomous AA (Below and Above Different Cut-offs)

				Unadjusted ordinal logistic regression for ROP severity			
	Descriptive data			OR (95% CI)			
Cut-off value for Mean AUC for AA	ROP severity	AA below cut-off n (%)	AA above cut-off n (%)	Impact of DHA for AA below cut-off on ROP severity	Impact of DHA for AA above cut-off on ROP severity	p-value for interaction	p-value for proportional odds assumption
**6.5**	**No ROP**	3 (33.3%)	68 (41.0%)	2.22 (0.24 - 20.19)	0.47 (0.34 - 0.66)	0.17	0.11
	**ROP Stage 1-2**	2 (22.2%)	52 (31.3%)				
	**ROP Stage 3 or treatment**	4 (44.4%)	46 (27.7%)				
**6.6**	**No ROP**	5 (38.5%)	66 (40.7%)	1.53 (0.41 - 5.75)	0.45 (0.32 - 0.64)	0.08	0.62
	**ROP Stage 1-2**	3 (23.1%)	51 (31.5%)				
	**ROP Stage 3 or treatment**	5 (38.5%)	45 (27.8%)				
**6.7**	**No ROP**	6 (40.0%)	65 (40.6%)	1.67 (0.44 - 6.25)	0.44 (0.31 - 0.63)	0.06	0.79
	**ROP Stage 1-2**	4 (26.7%)	50 (31.3%)				
	**ROP Stage 3 or treatment**	5 (33.3%)	45 (28.1%)				
**6.8**	**No ROP**	6 (37.5%)	65 (40.9%)	1.65 (0.44 - 6.16)	0.44 (0.31 - 0.62)	0.06	0.93
	**ROP Stage 1-2**	5 (31.3%)	49 (30.8%)				
	**ROP Stage 3 or treatment**	5 (31.3%)	45 (28.3%)				
**6.9**	**No ROP**	6 (37.5%)	65 (40.9%)	1.65 (0.44 - 6.16)	0.44 (0.31 - 0.62)	0.06	0.93
	**ROP Stage 1-2**	5 (31.3%)	49 (30.8%)				
	**ROP Stage 3 or treatment**	5 (31.3%)	45 (28.3%)				
**7**	**No ROP**	9 (45.0%)	62 (40.0%)	1.40 (0.42 - 4.63)	0.42 (0.29 - 0.60)	0.06	0.63
	**ROP Stage 1-2**	5 (25.0%)	49 (31.6%)				
	**ROP Stage 3 or treatment**	6 (30.0%)	44 (28.4%)				
**7.1**	**No ROP**	10 (47.6%)	61 (39.6%)	1.16 (0.36 - 3.72)	0.42 (0.29 - 0.60)	0.10	0.61
	**ROP Stage 1-2**	5 (23.8%)	49 (31.8%)				
	**ROP Stage 3 or treatment**	6 (28.6%)	44 (28.6%)				
**7.2**	**No ROP**	10 (43.5%)	61 (40.1%)	1.43 (0.51 - 4.06)	0.41 (0.29 - 0.59)	0.03	0.52
	**ROP Stage 1-2**	6 (26.1%)	48 (31.6%)				
	**ROP Stage 3 or treatment**	7 (30.4%)	43 (28.3%)				
**7.3**	**No ROP**	12 (41.4%)	59 (40.4%)	1.40 (0.56 - 3.52)	0.40 (0.27 - 0.58)	0.01	0.07
	**ROP Stage 1-2**	7 (24.1%)	47 (32.2%)				
	**ROP Stage 3 or treatment**	10 (34.5%)	40 (27.4%)				
**7.4**	**No ROP**	15 (39.5%)	56 (40.9%)	1.31 (0.53 - 3.24)	0.40 (0.27 - 0.60)	0.02	0.006
	**ROP Stage 1-2**	8 (21.1%)	46 (33.6%)				
	**ROP Stage 3 or treatment**	15 (39.5%)	35 (25.5%)				
**7.5**	**No ROP**	17 (37.8%)	54 (41.5%)	1.19 (0.50 - 2.81)	0.40 (0.26 - 0.60)	0.02	0.04
	**ROP Stage 1-2**	10 (22.2%)	44 (33.8%)				

© 2021 Hellström A et al. *JAMA Network Open.*

				Unadjusted ordinal logistic regression for ROP severity			
	Descriptive data			OR (95% CI)			
Cut-off value for Mean AUC for AA	ROP severity	AA below cut-off n (%)	AA above cut-off n (%)	Impact of DHA for AA below cut-off on ROP severity	Impact of DHA for AA above cut-off on ROP severity	p-value for interaction	p-value for proportional odds assumption
	**ROP Stage 3 or treatment**	18 (40.0%)	32 (24.6%)				
**7.6**	**No ROP**	18 (37.5%)	53 (41.7%)	1.12 (0.48 - 2.62)	0.40 (0.27 - 0.60)	0.03	0.04
	**ROP Stage 1-2**	11 (22.9%)	43 (33.9%)				
	**ROP Stage 3 or treatment**	19 (39.6%)	31 (24.4%)				
**7.7**	**No ROP**	22 (40.7%)	49 (40.5%)	0.96 (0.43 - 2.15)	0.39 (0.26 - 0.59)	0.05	0.01
	**ROP Stage 1-2**	12 (22.2%)	42 (34.7%)				
	**ROP Stage 3 or treatment**	20 (37.0%)	30 (24.8%)				
**7.8**	**No ROP**	23 (39.0%)	48 (41.4%)	0.74 (0.37 - 1.51)	0.40 (0.26 - 0.62)	0.14	0.0
	**ROP Stage 1-2**	14 (23.7%)	40 (34.5%)				
	**ROP Stage 3 or treatment**	22 (37.3%)	28 (24.1%)				
**7.9**	**No ROP**	25 (39.1%)	46 (41.4%)	0.70 (0.36 - 1.38)	0.40 (0.26 - 0.62)	0.17	0.07
	**ROP Stage 1-2**	16 (25.0%)	38 (34.2%)				
	**ROP Stage 3 or treatment**	23 (35.9%)	27 (24.3%)				
**8**	**No ROP**	26 (37.7%)	45 (42.5%)	0.67 (0.34 - 1.32)	0.40 (0.26 - 0.62)	0.20	0.20
	**ROP Stage 1-2**	19 (27.5%)	35 (33.0%)				
	**ROP Stage 3 or treatment**	24 (34.8%)	26 (24.5%)				
**8.1**	**No ROP**	29 (38.2%)	42 (42.4%)	0.60 (0.32 - 1.10)	0.41 (0.26 - 0.64)	0.34	0.26
	**ROP Stage 1-2**	21 (27.6%)	33 (33.3%)				
	**ROP Stage 3 or treatment**	26 (34.2%)	24 (24.2%)				
**8.2**	**No ROP**	30 (37.0%)	41 (43.6%)	0.57 (0.31 - 1.04)	0.42 (0.27 - 0.66)	0.45	0.32
	**ROP Stage 1-2**	23 (28.4%)	31 (33.0%)				
	**ROP Stage 3 or treatment**	28 (34.6%)	22 (23.4%)				
**8.3**	**No ROP**	31 (36.9%)	40 (44.0%)	0.53 (0.29 - 0.98)	0.43 (0.27 - 0.68)	0.57	0.40
	**ROP Stage 1-2**	24 (28.6%)	30 (33.0%)				
	**ROP Stage 3 or treatment**	29 (34.5%)	21 (23.1%)				
**8.4**	**No ROP**	37 (40.2%)	34 (41.0%)	0.40 (0.23 - 0.72)	0.45 (0.28 - 0.71)	0.79	0.31
	**ROP Stage 1-2**	25 (27.2%)	29 (34.9%)				
	**ROP Stage 3 or treatment**	30 (32.6%)	20 (24.1%)				
**8.5**	**No ROP**	38 (40.0%)	33 (41.3%)	0.38 (0.21 - 0.67)	0.45 (0.28 - 0.74)	0.62	0.35
	**ROP Stage 1-2**	26 (27.4%)	28 (35.0%)				
	**ROP Stage 3 or treatment**	31 (32.6%)	19 (23.8%)				
**8.6**	**No ROP**	39 (39.0%)	32 (42.7%)	0.41 (0.23 - 0.72)	0.46 (0.28 - 0.76)	0.74	0.29
	**ROP Stage 1-2**	27 (27.0%)	27 (36.0%)				
	**ROP Stage 3 or treatment**	34 (34.0%)	16 (21.3%)				
**8.7**	**No ROP**	41 (38.7%)	30 (43.5%)	0.40 (0.24 - 0.69)	0.45 (0.27 - 0.75)	0.79	0.72
	**ROP Stage 1-2**	31 (29.2%)	23 (33.3%)				

© 2021 Hellström A et al. *JAMA Network Open.*

				Unadjusted ordinal logistic regression for ROP severity			
	Descriptive data			OR (95% CI)			
Cut-off value for Mean AUC for AA	ROP severity	AA below cut-off n (%)	AA above cut-off n (%)	Impact of DHA for AA below cut-off on ROP severity	Impact of DHA for AA above cut-off on ROP severity	p-value for interaction	p-value for proportional odds assumption
	**ROP Stage 3 or treatment**	34 (32.1%)	16 (23.2%)				
**8.8**	**No ROP**	42 (38.2%)	29 (44.6%)	0.40 (0.24 - 0.69)	0.42 (0.24 - 0.73)	0.92	0.89
	**ROP Stage 1-2**	33 (30.0%)	21 (32.3%)				
	**ROP Stage 3 or treatment**	35 (31.8%)	15 (23.1%)				
**8.9**	**No ROP**	43 (37.7%)	28 (45.9%)	0.45 (0.28 - 0.74)	0.40 (0.22 - 0.73)	0.75	0.69
	**ROP Stage 1-2**	35 (30.7%)	19 (31.1%)				
	**ROP Stage 3 or treatment**	36 (31.6%)	14 (23.0%)				
**9**	**No ROP**	44 (37.0%)	27 (48.2%)	0.48 (0.30 - 0.78)	0.43 (0.23 - 0.79)	0.76	0.58
	**ROP Stage 1-2**	36 (30.3%)	18 (32.1%)				
	**ROP Stage 3 or treatment**	39 (32.8%)	11 (19.6%)				
**9.1**	**No ROP**	45 (37.2%)	26 (48.1%)	0.50 (0.32 - 0.80)	0.39 (0.21 - 0.75)	0.54	0.42
	**ROP Stage 1-2**	37 (30.6%)	17 (31.5%)				
	**ROP Stage 3 or treatment**	39 (32.2%)	11 (20.4%)				
**9.2**	**No ROP**	49 (38.3%)	22 (46.8%)	0.48 (0.31 - 0.74)	0.43 (0.22 - 0.83)	0.78	0.39
	**ROP Stage 1-2**	38 (29.7%)	16 (34.0%)				
	**ROP Stage 3 or treatment**	41 (32.0%)	9 (19.1%)				
**9.3**	**No ROP**	52 (38.8%)	19 (46.3%)	0.50 (0.33 - 0.75)	0.36 (0.17 - 0.76)	0.45	0.26
	**ROP Stage 1-2**	41 (30.6%)	13 (31.7%)				
	**ROP Stage 3 or treatment**	41 (30.6%)	9 (22.0%)				
**9.4**	**No ROP**	53 (38.4%)	18 (48.6%)	0.51 (0.34 - 0.75)	0.35 (0.16 - 0.78)	0.41	0.41
	**ROP Stage 1-2**	43 (31.2%)	11 (29.7%)				
	**ROP Stage 3 or treatment**	42 (30.4%)	8 (21.6%)				
**9.5**	**No ROP**	53 (37.1%)	18 (56.3%)	0.52 (0.35 - 0.77)	0.32 (0.13 - 0.80)	0.34	0.70
	**ROP Stage 1-2**	47 (32.9%)	7 (21.9%)				
	**ROP Stage 3 or treatment**	43 (30.1%)	7 (21.9%)				
**9.6**	**No ROP**	54 (37.5%)	17 (54.8%)	0.51 (0.35 - 0.75)	0.34 (0.14 - 0.85)	0.41	0.72
	**ROP Stage 1-2**	47 (32.6%)	7 (22.6%)				
	**ROP Stage 3 or treatment**	43 (29.9%)	7 (22.6%)				
**9.7**	**No ROP**	54 (37.0%)	17 (58.6%)	0.54 (0.37 - 0.78)	0.28 (0.10 - 0.77)	0.23	0.18
	**ROP Stage 1-2**	49 (33.6%)	5 (17.2%)				
	**ROP Stage 3 or treatment**	43 (29.5%)	7 (24.1%)				
**9.8**	**No ROP**	54 (36.5%)	17 (63.0%)	0.55 (0.38 - 0.79)	0.29 (0.11 - 0.81)	0.25	0.14
	**ROP Stage 1-2**	50 (33.8%)	4 (14.8%)				
	**ROP Stage 3 or treatment**	44 (29.7%)	6 (22.2%)				
**9.9**	**No ROP**	55 (36.7%)	16 (64.0%)	0.54 (0.37 - 0.77)	0.36 (0.13 - 1.00)	0.47	0.19
	**ROP Stage 1-2**	50 (33.3%)	4 (16.0%)				

© 2021 Hellström A et al. *JAMA Network Open.*

				Unadjusted ordinal logistic regression for ROP severity			
	Descriptive data			OR (95% CI)			
Cut-off value for Mean AUC for AA	ROP severity	AA below cut-off n (%)	AA above cut-off n (%)	Impact of DHA for AA below cut-off on ROP severity	Impact of DHA for AA above cut-off on ROP severity	p-value for interaction	p-value for proportional odds assumption
	**ROP Stage 3 or treatment**	45 (30.0%)	5 (20.0%)				
**10**	**No ROP**	57 (37.5%)	14 (60.9%)	0.52 (0.36 - 0.75)	0.35 (0.13 - 0.95)	0.45	0.21
	**ROP Stage 1-2**	50 (32.9%)	4 (17.4%)				
	**ROP Stage 3 or treatment**	45 (29.6%)	5 (21.7%)				
**10.1**	**No ROP**	60 (38.2%)	11 (61.1%)	0.53 (0.38 - 0.76)	0.26 (0.08 - 0.86)	0.26	0.53
	**ROP Stage 1-2**	51 (32.5%)	3 (16.7%)				
	**ROP Stage 3 or treatment**	46 (29.3%)	4 (22.2%)				
**10.2**	**No ROP**	60 (37.7%)	11 (68.8%)	0.55 (0.39 - 0.77)	0.20 (0.04 - 1.04)	0.24	0.90
	**ROP Stage 1-2**	51 (32.1%)	3 (18.8%)				
	**ROP Stage 3 or treatment**	48 (30.2%)	2 (12.5%)				
**10.3**	**No ROP**	62 (38.5%)	9 (64.3%)	0.53 (0.38 - 0.74)	0.24 (0.05 - 1.19)	0.34	0.92
	**ROP Stage 1-2**	51 (31.7%)	3 (21.4%)				
	**ROP Stage 3 or treatment**	48 (29.8%)	2 (14.3%)				
**10.4**	**No ROP**	62 (38.5%)	9 (64.3%)	0.53 (0.38 - 0.74)	0.24 (0.05 - 1.19)	0.34	0.92
	**ROP Stage 1-2**	51 (31.7%)	3 (21.4%)				
	**ROP Stage 3 or treatment**	48 (29.8%)	2 (14.3%)				
**10.5**	**No ROP**	64 (39.3%)	7 (58.3%)	0.51 (0.37 - 0.72)	0.29 (0.06 - 1.45)	0.50	0.94
	**ROP Stage 1-2**	51 (31.3%)	3 (25.0%)				
	**ROP Stage 3 or treatment**	48 (29.4%)	2 (16.7%)				

ROP = retinopathy of prematurity; LCPUFAs = long-chain polyunsaturated fatty acid; OR = odds ratio; CI = confidence interval; AUC = area under the curve; AA = arachidonic acid; DHA = docosahexaenoic acid

Both AA and DHA were calculated as mean AUC days 1-28

These analyses were exploratory, and hypothesis generating, with the interaction interpreted at 0.10 level and the proportional odds assumption at 0.05 level.

© 2021 Hellström A et al. *JAMA Network Open.*

eTable 3. Gestational Age and Birth Weight Adjusted Ordinal Logistic Regression for ROP Severity Studying Interaction Between Continuous DHA and Dichotomous AA (Below and Above Different Cut-offs)

				Adjusted ordinal logistic regression for ROP severity			
	Descriptive data			OR (95% CI)			
Cut-off value for Mean AUC for AA	ROP severity	AA below cut-off n (%)	AA above cut-off n (%)	Impact of DHA for AA below cut-off on ROP severity	Impact of DHA for AA above cut-off on ROP severity	p-value for interaction	p-value for proportional odds assumption
**6.5**	**No ROP**	3 (33.3%)	68 (41.0%)	1.99 (0.21 - 18.48)	0.65 (0.45 - 0.94)	0.33	0.05
	**ROP Stage 1-2**	2 (22.2%)	52 (31.3%)				
	**ROP Stage 3 or treatment**	4 (44.4%)	46 (27.7%)				
**6.6**	**No ROP**	5 (38.5%)	66 (40.7%)	1.71 (0.44 - 6.60)	0.64 (0.44 - 0.94)	0.17	0.18
	**ROP Stage 1-2**	3 (23.1%)	51 (31.5%)				
	**ROP Stage 3 or treatment**	5 (38.5%)	45 (27.8%)				
**6.7**	**No ROP**	6 (40.0%)	65 (40.6%)	1.93 (0.50 - 7.40)	0.62 (0.42 - 0.92)	0.11	0.19
	**ROP Stage 1-2**	4 (26.7%)	50 (31.3%)				
	**ROP Stage 3 or treatment**	5 (33.3%)	45 (28.1%)				
**6.8**	**No ROP**	6 (37.5%)	65 (40.9%)	1.84 (0.48 - 7.03)	0.63 (0.43 - 0.93)	0.13	0.16
	**ROP Stage 1-2**	5 (31.3%)	49 (30.8%)				
	**ROP Stage 3 or treatment**	5 (31.3%)	45 (28.3%)				
**6.9**	**No ROP**	6 (37.5%)	65 (40.9%)	1.84 (0.48 - 7.03)	0.63 (0.43 - 0.93)	0.13	0.16
	**ROP Stage 1-2**	5 (31.3%)	49 (30.8%)				
	**ROP Stage 3 or treatment**	5 (31.3%)	45 (28.3%)				
**7**	**No ROP**	9 (45.0%)	62 (40.0%)	1.70 (0.49 - 5.85)	0.60 (0.40 - 0.90)	0.12	0.18
	**ROP Stage 1-2**	5 (25.0%)	49 (31.6%)				
	**ROP Stage 3 or treatment**	6 (30.0%)	44 (28.4%)				
**7.1**	**No ROP**	10 (47.6%)	61 (39.6%)	1.50 (0.45 - 5.02)	0.60 (0.40 - 0.90)	0.16	0.18
	**ROP Stage 1-2**	5 (23.8%)	49 (31.8%)				
	**ROP Stage 3 or treatment**	6 (28.6%)	44 (28.6%)				
**7.2**	**No ROP**	10 (43.5%)	61 (40.1%)	1.40 (0.47 - 4.17)	0.60 (0.40 - 0.90)	0.15	0.17
	**ROP Stage 1-2**	6 (26.1%)	48 (31.6%)				
	**ROP Stage 3 or treatment**	7 (30.4%)	43 (28.3%)				
**7.3**	**No ROP**	12 (41.4%)	59 (40.4%)	1.39 (0.53 - 3.64)	0.60 (0.40 - 0.92)	0.12	0.06
	**ROP Stage 1-2**	7 (24.1%)	47 (32.2%)				
	**ROP Stage 3 or treatment**	10 (34.5%)	40 (27.4%)				
**7.4**	**No ROP**	15 (39.5%)	56 (40.9%)	1.39 (0.54 - 3.56)	0.62 (0.40 - 0.95)	0.13	0.02
	**ROP Stage 1-2**	8 (21.1%)	46 (33.6%)				
	**ROP Stage 3 or treatment**	15 (39.5%)	35 (25.5%)				
**7.5**	**No ROP**	17 (37.8%)	54 (41.5%)	1.40 (0.56 - 3.46)	0.63 (0.40 - 0.98)	0.12	0.05
	**ROP Stage 1-2**	10 (22.2%)	44 (33.8%)				

© 2021 Hellström A et al. *JAMA Network Open.*

				Adjusted ordinal logistic regression for ROP severity			
	Descriptive data			OR (95% CI)			
Cut-off value for Mean AUC for AA	ROP severity	AA below cut-off n (%)	AA above cut-off n (%)	Impact of DHA for AA below cut-off on ROP severity	Impact of DHA for AA above cut-off on ROP severity	p-value for interaction	p-value for proportional odds assumption
	**ROP Stage 3 or treatment**	18 (40.0%)	32 (24.6%)				
**7.6**	**No ROP**	18 (37.5%)	53 (41.7%)	1.35 (0.55 - 3.31)	0.64 (0.41 - 1.00)	0.14	0.05
	**ROP Stage 1-2**	11 (22.9%)	43 (33.9%)				
	**ROP Stage 3 or treatment**	19 (39.6%)	31 (24.4%)				
**7.7**	**No ROP**	22 (40.7%)	49 (40.5%)	1.16 (0.49 - 2.73)	0.62 (0.39 - 0.98)	0.20	0.03
	**ROP Stage 1-2**	12 (22.2%)	42 (34.7%)				
	**ROP Stage 3 or treatment**	20 (37.0%)	30 (24.8%)				
**7.8**	**No ROP**	23 (39.0%)	48 (41.4%)	0.75 (0.36 - 1.58)	0.71 (0.44 - 1.13)	0.90	0.06
	**ROP Stage 1-2**	14 (23.7%)	40 (34.5%)				
	**ROP Stage 3 or treatment**	22 (37.3%)	28 (24.1%)				
**7.9**	**No ROP**	25 (39.1%)	46 (41.4%)	0.71 (0.35 - 1.45)	0.69 (0.43 - 1.10)	0.93	0.07
	**ROP Stage 1-2**	16 (25.0%)	38 (34.2%)				
	**ROP Stage 3 or treatment**	23 (35.9%)	27 (24.3%)				
**8**	**No ROP**	26 (37.7%)	45 (42.5%)	0.72 (0.35 - 1.46)	0.70 (0.43 - 1.13)	0.95	0.13
	**ROP Stage 1-2**	19 (27.5%)	35 (33.0%)				
	**ROP Stage 3 or treatment**	24 (34.8%)	26 (24.5%)				
**8.1**	**No ROP**	29 (38.2%)	42 (42.4%)	0.64 (0.34 - 1.23)	0.72 (0.44 - 1.18)	0.78	0.14
	**ROP Stage 1-2**	21 (27.6%)	33 (33.3%)				
	**ROP Stage 3 or treatment**	26 (34.2%)	24 (24.2%)				
**8.2**	**No ROP**	30 (37.0%)	41 (43.6%)	0.63 (0.33 - 1.19)	0.69 (0.42 - 1.14)	0.81	0.13
	**ROP Stage 1-2**	23 (28.4%)	31 (33.0%)				
	**ROP Stage 3 or treatment**	28 (34.6%)	22 (23.4%)				
**8.3**	**No ROP**	31 (36.9%)	40 (44.0%)	0.59 (0.31 - 1.12)	0.71 (0.43 - 1.18)	0.66	0.12
	**ROP Stage 1-2**	24 (28.6%)	30 (33.0%)				
	**ROP Stage 3 or treatment**	29 (34.5%)	21 (23.1%)				
**8.4**	**No ROP**	37 (40.2%)	34 (41.0%)	0.49 (0.26 - 0.90)	0.74 (0.44 - 1.24)	0.31	0.09
	**ROP Stage 1-2**	25 (27.2%)	29 (34.9%)				
	**ROP Stage 3 or treatment**	30 (32.6%)	20 (24.1%)				
**8.5**	**No ROP**	38 (40.0%)	33 (41.3%)	0.47 (0.25 - 0.87)	0.74 (0.44 - 1.27)	0.26	0.09
	**ROP Stage 1-2**	26 (27.4%)	28 (35.0%)				
	**ROP Stage 3 or treatment**	31 (32.6%)	19 (23.8%)				
**8.6**	**No ROP**	39 (39.0%)	32 (42.7%)	0.51 (0.28 - 0.92)	0.75 (0.44 - 1.29)	0.33	0.07
	**ROP Stage 1-2**	27 (27.0%)	27 (36.0%)				
	**ROP Stage 3 or treatment**	34 (34.0%)	16 (21.3%)				
**8.7**	**No ROP**	41 (38.7%)	30 (43.5%)	0.49 (0.28 - 0.87)	0.74 (0.42 - 1.31)	0.31	0.14
	**ROP Stage 1-2**	31 (29.2%)	23 (33.3%)				

© 2021 Hellström A et al. *JAMA Network Open.*

				Adjusted ordinal logistic regression for ROP severity			
	Descriptive data			OR (95% CI)			
Cut-off value for Mean AUC for AA	ROP severity	AA below cut-off n (%)	AA above cut-off n (%)	Impact of DHA for AA below cut-off on ROP severity	Impact of DHA for AA above cut-off on ROP severity	p-value for interaction	p-value for proportional odds assumption
	**ROP Stage 3 or treatment**	34 (32.1%)	16 (23.2%)				
**8.8**	**No ROP**	42 (38.2%)	29 (44.6%)	0.52 (0.29 - 0.91)	0.65 (0.35 - 1.21)	0.58	0.17
	**ROP Stage 1-2**	33 (30.0%)	21 (32.3%)				
	**ROP Stage 3 or treatment**	35 (31.8%)	15 (23.1%)				
**8.9**	**No ROP**	43 (37.7%)	28 (45.9%)	0.61 (0.36 - 1.04)	0.59 (0.30 - 1.13)	0.92	0.14
	**ROP Stage 1-2**	35 (30.7%)	19 (31.1%)				
	**ROP Stage 3 or treatment**	36 (31.6%)	14 (23.0%)				
**9**	**No ROP**	44 (37.0%)	27 (48.2%)	0.62 (0.37 - 1.04)	0.68 (0.35 - 1.33)	0.83	0.09
	**ROP Stage 1-2**	36 (30.3%)	18 (32.1%)				
	**ROP Stage 3 or treatment**	39 (32.8%)	11 (19.6%)				
**9.1**	**No ROP**	45 (37.2%)	26 (48.1%)	0.65 (0.40 - 1.07)	0.62 (0.31 - 1.24)	0.91	0.05
	**ROP Stage 1-2**	37 (30.6%)	17 (31.5%)				
	**ROP Stage 3 or treatment**	39 (32.2%)	11 (20.4%)				
**9.2**	**No ROP**	49 (38.3%)	22 (46.8%)	0.64 (0.40 - 1.02)	0.70 (0.34 - 1.42)	0.82	0.05
	**ROP Stage 1-2**	38 (29.7%)	16 (34.0%)				
	**ROP Stage 3 or treatment**	41 (32.0%)	9 (19.1%)				
**9.3**	**No ROP**	52 (38.8%)	19 (46.3%)	0.66 (0.43 - 1.03)	0.59 (0.26 - 1.32)	0.79	0.03
	**ROP Stage 1-2**	41 (30.6%)	13 (31.7%)				
	**ROP Stage 3 or treatment**	41 (30.6%)	9 (22.0%)				
**9.4**	**No ROP**	53 (38.4%)	18 (48.6%)	0.70 (0.45 - 1.07)	0.49 (0.20 - 1.19)	0.47	0.04
	**ROP Stage 1-2**	43 (31.2%)	11 (29.7%)				
	**ROP Stage 3 or treatment**	42 (30.4%)	8 (21.6%)				
**9.5**	**No ROP**	53 (37.1%)	18 (56.3%)	0.71 (0.47 - 1.09)	0.45 (0.17 - 1.20)	0.39	0.10
	**ROP Stage 1-2**	47 (32.9%)	7 (21.9%)				
	**ROP Stage 3 or treatment**	43 (30.1%)	7 (21.9%)				
**9.6**	**No ROP**	54 (37.5%)	17 (54.8%)	0.69 (0.46 - 1.05)	0.47 (0.17 - 1.30)	0.48	0.11
	**ROP Stage 1-2**	47 (32.6%)	7 (22.6%)				
	**ROP Stage 3 or treatment**	43 (29.9%)	7 (22.6%)				
**9.7**	**No ROP**	54 (37.0%)	17 (58.6%)	0.73 (0.49 - 1.10)	0.35 (0.11 - 1.07)	0.22	0.01
	**ROP Stage 1-2**	49 (33.6%)	5 (17.2%)				
	**ROP Stage 3 or treatment**	43 (29.5%)	7 (24.1%)				
**9.8**	**No ROP**	54 (36.5%)	17 (63.0%)	0.73 (0.49 - 1.09)	0.36 (0.12 - 1.10)	0.24	0.010
	**ROP Stage 1-2**	50 (33.8%)	4 (14.8%)				
	**ROP Stage 3 or treatment**	44 (29.7%)	6 (22.2%)				
**9.9**	**No ROP**	55 (36.7%)	16 (64.0%)	0.70 (0.47 - 1.05)	0.44 (0.14 - 1.34)	0.43	0.02
	**ROP Stage 1-2**	50 (33.3%)	4 (16.0%)				

© 2021 Hellström A et al. *JAMA Network Open.*

				Adjusted ordinal logistic regression for ROP severity			
	Descriptive data			OR (95% CI)			
Cut-off value for Mean AUC for AA	ROP severity	AA below cut-off n (%)	AA above cut-off n (%)	Impact of DHA for AA below cut-off on ROP severity	Impact of DHA for AA above cut-off on ROP severity	p-value for interaction	p-value for proportional odds assumption
	**ROP Stage 3 or treatment**	45 (30.0%)	5 (20.0%)				
**10**	**No ROP**	57 (37.5%)	14 (60.9%)	0.69 (0.46 - 1.03)	0.42 (0.13 - 1.29)	0.41	0.02
	**ROP Stage 1-2**	50 (32.9%)	4 (17.4%)				
	**ROP Stage 3 or treatment**	45 (29.6%)	5 (21.7%)				
**10.1**	**No ROP**	60 (38.2%)	11 (61.1%)	0.72 (0.49 - 1.05)	0.28 (0.07 - 1.12)	0.19	0.06
	**ROP Stage 1-2**	51 (32.5%)	3 (16.7%)				
	**ROP Stage 3 or treatment**	46 (29.3%)	4 (22.2%)				
**10.2**	**No ROP**	60 (37.7%)	11 (68.8%)	0.75 (0.52 - 1.09)	0.16 (0.03 - 1.04)	0.11	0.13
	**ROP Stage 1-2**	51 (32.1%)	3 (18.8%)				
	**ROP Stage 3 or treatment**	48 (30.2%)	2 (12.5%)				
**10.3**	**No ROP**	62 (38.5%)	9 (64.3%)	0.72 (0.50 - 1.05)	0.20 (0.03 - 1.22)	0.17	0.15
	**ROP Stage 1-2**	51 (31.7%)	3 (21.4%)				
	**ROP Stage 3 or treatment**	48 (29.8%)	2 (14.3%)				
**10.4**	**No ROP**	62 (38.5%)	9 (64.3%)	0.72 (0.50 - 1.05)	0.20 (0.03 - 1.22)	0.17	0.15
	**ROP Stage 1-2**	51 (31.7%)	3 (21.4%)				
	**ROP Stage 3 or treatment**	48 (29.8%)	2 (14.3%)				
**10.5**	**No ROP**	64 (39.3%)	7 (58.3%)	0.71 (0.49 - 1.02)	0.23 (0.04 - 1.44)	0.24	0.16
	**ROP Stage 1-2**	51 (31.3%)	3 (25.0%)				
	**ROP Stage 3 or treatment**	48 (29.4%)	2 (16.7%)				

ROP = retinopathy of prematurity; LCPUFAs = long-chain polyunsaturated fatty acid; OR = odds ratio; CI = confidence interval; AUC = area under the curve; AA = arachidonic acid; DHA = docosahexaenoic acid

Both LCPUFAs calculated as mean AUC days 1-28

These analyses were exploratory, and hypothesis generating, with the interaction interpreted at 0.10 level and the proportional odds assumption at 0.05 level.

© 2021 Hellström A et al. *JAMA Network Open.*

eFigure. Gestational Age and Birth Weight Adjusted Odds-Ratios Obtained from the Ordinal Logistic Regression for ROP Severity Studying Interaction Between Continuous DHA and Dichotomous AA (Below and Above Different Cut-offs)

The adjusted odds ratios in the analyses relates to an impact of an increase of 0.5 mol% of DHA for different (AA) (20:4 ω-6) cut-offs. Each cut-off value of AA mol% corresponds to two analyses, one including infants having AA proportion below the cut-off (seen in red) and one with infants having AA proportion above the cut-off (seen in blue). An OR of 0.4 indicates a 60% decrease in log-odds for ROP progression between ROP stage 3 or treatment to ROP stage 1 or 2 and between ROP stage 1 or 2 to no ROP.

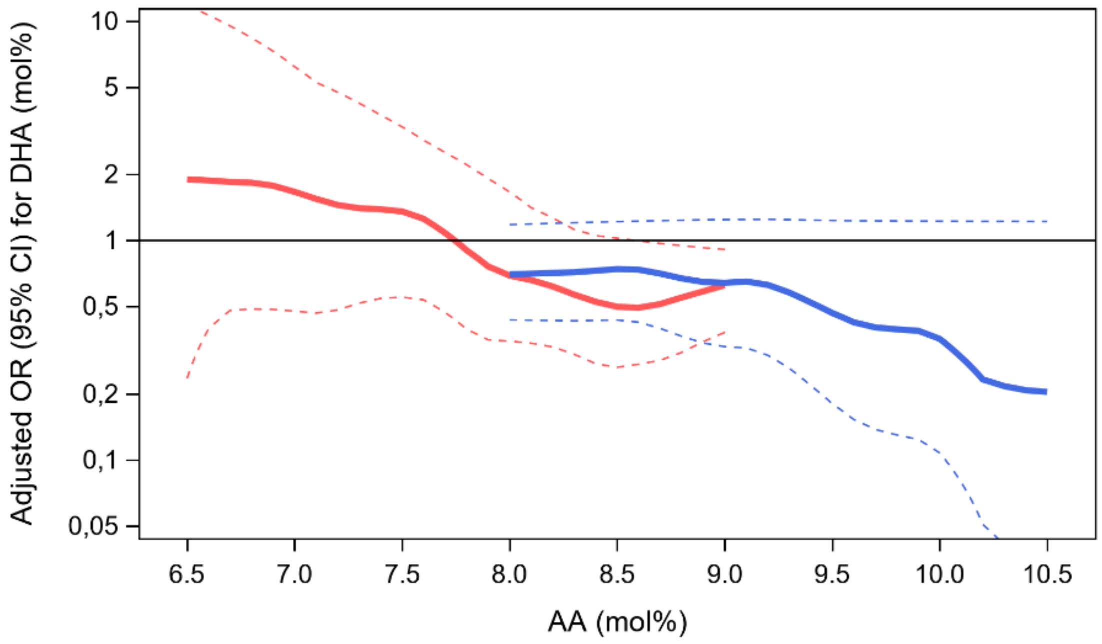

© 2021 Hellström A et al. *JAMA Network Open.*
